# Melatonin Preserves Mitochondrial Redox Balance in UVB-Exposed Human Keratinocytes

**DOI:** 10.3390/antiox15080939

**Published:** 2026-07-29

**Authors:** Mayuko Okura, Kento Takaya, Tatsuyuki Ishii, Maciej Gagat, Agnieszka Wolnicka-Glubisz, Kazuo Kishi, Keisuke Okabe, Russel J. Reiter, Andrzej T. Slominski, Konrad Kleszczyński

**Affiliations:** 1 Department of Plastic and Reconstructive Surgery, Keio University School of Medicine, 35 Shinanomachi, Shinjuku-ku, Tokyo 160-8582, Japan; mayuko.okura@keio.jp (M.O.); kentotakaya@keio.jp (K.T.); ttsyksh@keio.jp (T.I.); kkishi@keio.jp (K.K.); dawndawn@keio.jp (K.O.); 2 Department of Histology and Embryology, Faculty of Medicine, Nicolaus Copernicus University in Toruń, Collegium Medicum in Bydgoszcz, Karłowicza 24, 85-092 Bydgoszcz, Poland; mgagat@cm.umk.pl; 3 Department of Biophysics and Cancer Biology, Faculty of Biochemistry, Biophysics and Biotechnology, Jagiellonian University, Gronostajowa 7, 30-387 Krakow, Poland; a.wolnicka-glubisz@uj.edu.pl; 4 Department of Cell Systems and Anatomy, UT Health San Antonio, Long School of Medicine, San Antonio, TX 78229, USA; reiter@uthscsa.edu; 5 Department of Dermatology, Comprehensive Cancer Center, University of Alabama at Birmingham, Birmingham, AL 35294, USA; aslominski@uabmc.edu; 6 Pathology and Laboratory Medicine Service, VA Medical Center, Birmingham, AL 35294, USA; 7 Department of Dermatology, University of Münster, Von-Esmarch-Str. 58, 48149 Münster, Germany

**Keywords:** melatonin, mitochondrial homeostasis, oxidative stress, UVB radiation, primary keratinocytes

## Abstract

Melatonin (*N*-acetyl-5-methoxytryptamine) is an evolutionarily conserved indoleamine with potent antioxidant and cytoprotective properties. Due to its amphiphilic nature, melatonin readily accumulates in mitochondria, where it regulates redox homeostasis and bioenergetic function. Although melatonin has been implicated in cutaneous protection against ultraviolet radiation (UVR), the mitochondrial mechanisms underlying its effects in UVB-exposed keratinocytes remain insufficiently characterized. In this study, we investigated the mitochondrial-centered protective effects of melatonin in human epidermal keratinocytes (NHEKs) subjected to UVB irradiation. UVB exposure induced a dose-dependent decrease in cell viability and proliferation, accompanied by excessive reactive oxygen species (ROS) production, mitochondrial membrane depolarization (ΔΨm), ATP depletion, caspase-3 activation, and increased expression of DNA damage- and senescence-associated markers, including γH2AX, p53, and p16^INK4a^. Pre-treatment with melatonin attenuated these alterations by reducing oxidative stress, preserving ΔΨm, maintaining ATP production, suppressing apoptotic signaling, and modulating DNA damage- and senescence-related pathways. Collectively, our findings suggest that mitochondria are a central target of melatonin in UVB-exposed keratinocytes and demonstrate that maintenance of mitochondrial redox balance and bioenergetic integrity is associated with reduced apoptotic and senescence-related changes.

## 1. Introduction

External exposure to solar ultraviolet radiation (UVR) causes acute photodamage, premature aging, and skin cancer, processes driven by a combination of genotoxic, oxidative, neuroendocrine, and inflammatory stress [1,2,3,4,5,6]. The UVR spectrum comprises UVC (200–280 nm), UVB (280–315 nm), and UVA (315–400 nm). While UVC is fully absorbed by the ozone layer, UVA penetrates deeply into the dermis [7]. Highly energetic UVB (280–320 nm), which is absorbed in the upper epidermis [7,8], inflicts significant cellular damage. It induces direct DNA lesions such as cyclobutane pyrimidine dimers (CPDs), pyrimidine (6-4) pyrimidone photoproducts (6-4PPs), and double-strand breaks as well as secondary oxidative stress and mitochondrial dysfunction in cutaneous cells [9]. Prolonged UVR exposure leads to photoaging, characterized by wrinkles, skin laxity, hyperpigmentation, loss of elasticity, and in severe cases, contributes to photocarcinogenesis [10]. Notably, an estimated 90% of skin cancers are attributable to chronic UV exposure and photoaging, making it a major global health and economic burden [11]. Photoaging disrupts extracellular matrix components such as collagen and elastin and promotes persistent DNA damage, oxidative stress, and inflammation [12,13,14]. These changes share features with intrinsic aging, which arises from proliferative exhaustion, telomere attrition, and genomic instability, but extrinsic stressors—particularly UVA/UVB irradiation, pollution, and tobacco smoke—accelerate cutaneous decline. Both intrinsic and extrinsic factors converge on mitochondrial dysfunction and activation of DNA damage response pathways in skin cells [15,16,17]. A key early event is depolarization of the mitochondrial membrane potential (ΔΨm), a hallmark of both intrinsic and photo-induced aging [18,19].

Mitochondrial-derived reactive oxygen species (ROS) are central mediators of UV-induced damage. Persistent mtROS generation can amplify cellular dysfunction by promoting paracrine senescence [20] and sustaining DNA damage responses that enforce senescence programs [21,22,23]. Impaired mitochondrial turnover further contributes to senescence progression: delayed clearance of damaged mitochondria enhances the senescence-associated secretory phenotype (SASP) while maintaining cell-cycle arrest [24], a process termed mitochondrial dysfunction–associated senescence (MiDAS) [25]. Thus, strategies that reinforce mitochondrial function and bolster antioxidant capacity represent promising approaches to mitigate UV-induced cellular stress.

Melatonin (*N*-acetyl-5-methoxytryptamine) is an evolutionarily conserved indoleamine with multifunctional activities, including circadian regulation and coordination of cellular stress responses essential for survival and homeostasis [26,27,28,29,30,31]. While classically produced by the pineal gland under suprachiasmatic control, melatonin is also synthesized in peripheral organs, including the skin [26,28,29,30,31]. Three metabolic pathways—classical, indolic, and kynuric—operate cutaneously [31], generating melatonin metabolites such as 6-hydroxymelatonin, 5-methoxytryptamine, *N*^1^-acetyl-*N*^2^-formyl-5-methoxykynuramine (AFMK), and *N*^1^-acetyl-5-methoxykynuramine (AMK), which accumulate in the epidermis [31,32,33,34]. Melatonin is highly lipophilic and readily crosses cellular membranes, reaching intracellular compartments, including mitochondria, where its concentrations surpass those in blood [35,36]. Within mitochondria, melatonin interacts with lipid bilayers, stabilizes the inner membrane, and protects against oxidative damage [37,38]. Melatonin’s effects are mediated partly through its G-protein-coupled receptors MT1 and MT2, which can form heterodimers [31], though receptor-independent actions such as interactions with intracellular proteins including quinone reductase 2 have also been demonstrated [39]. Owing to these anti-oxidative, anti-inflammatory, and metabolic actions, melatonin has been proposed as a cytoprotective and potential anti-aging molecule [31,40]. Although both receptor-dependent and receptor-independent mechanisms have been implicated in melatonin signaling, the present study was designed to investigate the overall mitochondrial and cytoprotective responses to melatonin following UVB exposure rather than to dissect the relative contribution of individual signaling pathways.

Based on previous reports demonstrating endogenous melatonin production in the skin and its local tissue concentrations [31,32,33,34,35,36,37], the concentration range selected in the present study encompasses both physiologically relevant (10^−9^–10^−7^ M; 1–100 nM) and pharmacological (10^−3^ M; 1 mM) levels. While the lower concentrations more closely reflect tissue-relevant melatonin exposure, the highest concentration was intentionally included as a supraphysiological concentration to define the upper boundary of cytoprotective efficacy under conditions of severe UVB-induced oxidative stress.

In this study, we investigate the mitochondrial-centered protective actions of melatonin in human epidermal keratinocytes exposed to UVB. We focus on melatonin’s ability to preserve mitochondrial integrity, support cellular metabolism, modulate mitochondrial antioxidant capacity, and suppress apoptotic signaling. These mechanistic insights provide a crucial foundation for ongoing in vivo studies aimed at evaluating melatonin’s broader protective and potential anti-aging effects on skin physiology. By delineating how melatonin sustains mitochondrial resilience under UVB challenge, our work advances understanding of its role as an endogenous regulator of epidermal redox homeostasis.

## 2. Materials and Methods

### 2.1. Reagents

Melatonin (MEL), 1% penicillin-streptomycin solution (10,000 units of penicillin and 10 mg of streptomycin in 1 mL 0.9% NaCl), 0.05% trypsin/0.53 mM EDTA solution, 3-amino-7-dimethylamino-2-methyl-phenazine hydrochloride (Neutral Red, NR), 3-(4,5-dimethylthiazol-2-yl)-2,5-diphenyltetrazolium bromide (MTT), acetic acid, ethanol, HCl, isopropanol were purchased from Sigma (St. Louis, MO, USA).

### 2.2. Cell Culture, Melatonin Treatment, and UVB Exposure

Primary normal human epidermal keratinocytes (NHEKs; C-12006, passage 2, adult pooled donor, PromoCell, Heidelberg, Germany) were cultured in Keratinocyte Growth Medium 2 supplemented with 1% penicillin–streptomycin in a humidified atmosphere containing 5% CO_2_ at 37 °C. According to the manufacturer’s quality control, the cells were routinely tested and certified to be free of mycoplasma contamination. Prior to the start of the experimental treatment, cells were seeded and cultured for 24 h. Thereafter, the culture medium was replaced containing melatonin. Melatonin was initially dissolved in 20 μL of absolute ethanol and subsequently diluted with 980 μL phosphate-buffered saline (1×PBS, pH 7.4) to obtain a 10^−2^ M stock solution which was prepared fresh for every experiment. The stock solution was further diluted in culture medium to achieve the final experimental concentrations of 10^−3^ M (1 mM), 10^−5^ M (10 µM), 10^−7^ M (100 nM), and 10^−9^ M (1 nM), followed by a 1 h pre-incubation prior to UVB exposure, protected from light throughout preparation and handling. The corresponding final ethanol concentration in the culture medium did not exceed 0.2% (*v*/*v*). Briefly, cells were washed twice with 1×PBS to remove remnants of culture medium, and 1×PBS was added again before irradiation. Cells were exposed to UVB radiation using a Handheld UV Lamp, 6 W, UVM-57 (Analytik Jena Japan Co., Ltd., Yokohama, Japan). Prior to each series of experiments, the lamp was allowed to reach a stable operating output (warm-up). The delivered UVB dose was verified using a calibrated UV radiometer, and all irradiations were performed at a fixed lamp-to-sample distance under standardized experimental conditions. The spectral characteristics of the irradiation source were according to the manufacturer’s specifications. Cells were irradiated in a dose-dependent manner (10, 30, 50, 70, and 100 mJ/cm^2^), while sham-irradiated control cultures (0 mJ/cm^2^) were protected from UV exposure by covering the culture dishes with aluminium foil.

### 2.3. Cell Viability Assays

Cells were seeded on 96-well plates (0.2 × 10^5^ cells/well in the culture medium) and grown to subconfluence (as judged from light microscopy). Forty-eight hours after UVB irradiation, cell viability was assessed using the MTT assay according to the previously described procedure [41]. MTT (5 mg/mL in 1×PBS) was prepared with culture medium (the final dilution, 1:10), 100 μL of assay reagent was added to each well, cells were incubated for 3 h in a humidified atmosphere of 5% CO_2_ at 37 °C, and the resultant formazan crystals were dissolved using 100 μL isopropanol/0.04 N HCl.

In case of NR assay, cells 48 h post-UVR were incubated with NR solution (40 μg/mL in 1×PBS) dissolved 1:100 with culture medium as described earlier [42]. Briefly, 100 μL NR was added to each well of the plate, cells were incubated for 2 h in a humidified atmosphere of 5% CO_2_ at 37 °C. After that, cells were washed three times with 1×PBS and 150 μL of NR destain solution (1% acetic acid, 50% ethanol, 49% H_2_O) was added per well, and shaken for 10 min. The resultant plates were assessed using the Cytation5 microplate reader (BioTek Instruments, Inc., Winooski, VT, USA) at the optical density of λ = 570 nm and λ = 540 nm for MTT and NR, respectively while all the results were normalized to the control sample accordingly.

### 2.4. Crystal Violet Assessment

Adherent cell density was assessed using a ready-to-use crystal violet (CV) solution (Cell Biolabs, Inc., San Diego, CA, USA). Cells were seeded on 24-well plates (0.1 × 10^6^ cells/well) and allowed to reach approximately 70–80% confluence before treatment. Forty-eight hours after UVB irradiation, cells were washed twice with 1×PBS, stained with CV solution (0.1% in 10% ethanol) for 3 min at room temperature (RT), and remnants were washed again three times with 1×PBS. Plates were photographed and quantified using the Image J 1.51f software (National Institute of Health, Bethesda, MD, USA), and results were normalized to the control sample.

### 2.5. Oxidative Stress and Changes Within the Mitochondrial Transmembrane Potential (*ΔΨ*m)

Followed by desired time points, generation of oxidative stress and changes within the ΔΨm were assessed using DCFDA cellular ROS assay (Abcam, Cambridge, UK) and MitoPT^®^ TMRM assay (ImmunoChemistry, Davis, CA, USA) kits, respectively. Both assays were employed as an endpoint measurement in viable cells immediately after staining and washing. No time-lapse or real-time live-cell imaging was performed. Briefly, cells were seeded in cell chambers for microscopy or 96-well plates for fluorescence assessment (0.2 × 10^5^ cells/well in the culture medium). As a positive control for mitochondrial membrane depolarization, a separate group of non-irradiated keratinocytes was treated with 100 μM carbonyl cyanide m-chlorophenylhydrazone (CCCP) for 60 min at 37 °C immediately before TMRM staining. Next, cells were stained for 45 min at 37 °C with 20 μM DCFDA solution for ROS and 200 nM TMRM for ΔΨm. After that, cells were washed three times with 1×PBS and visualized using the Olympus EVIDENT inverted fluorescence microscope (Olympus, Westborough, MA, USA) or the Cytation5 microplate reader (BioTek Instruments, Inc.). Cells were evaluated with Ex./Em. = 485/535 nm for ROS and Ex./Em. = 540/570 nm for ΔΨm. Obtained results were presented as percentages of the control values.

### 2.6. Evaluation of ATP Synthesis

Alterations in mitochondrial homeostasis and resultant oxidative stress were assessed using luminescence assay for changes in ATP synthesis (Abcam, Cambridge, UK). Briefly, cells were seeded on 96-well plates (0.2 × 10^5^ cells/well). Followed by desired incubation time points, cells were treated by the assay buffers included in the kit, and reaction was determined by developer mix for 30 min (ATP assay) at RT in dark. The luminescence was determined using the Cytation5 microplate reader (BioTek Instruments, Inc.), and results were normalized and compared to the control sample.

### 2.7. Analysis of Cleavage of Caspases

Caspase 3 activity was assessed colorimetric along the manufacturer’s instruction (Abcam, Cambridge, UK). Cells (2 × 10^6^), followed by 48 h post-UVB, were resuspended in 50 μL of cooled lysis buffer and incubated on ice for 10 min, centrifuged at 10,000× *g* for 3 min, and the cytosolic fraction was taken for the assessment. Resultant sample was mixed with the reaction buffer/DTT, then 5 μL of the 4 mM DEVD-pNA substrate (200 μM final concentration) was added to each sample well, mixed well and incubated at 37 °C for 120 min. Finally, the optical density was measured at the λ = 405 nm using the Cytation5 microplate reader. The results were normalized and compared to the control sample.

### 2.8. Western Blotting Assessment

Total protein was extracted from cultured cells using the EzRIPA Lysis Kit (ATTO, Tokyo, Japan) following the manufacturer’s instructions. The EzRIPA lysis buffer was supplemented with the protease and phosphatase inhibitor cocktails supplied with the kit immediately before use. After washing with ice-cold PBS, cells were collected using a chilled cell scraper, snap-frozen in liquid nitrogen, briefly homogenized using an electric homogenizer, and subsequently incubated in lysis buffer for 2 h at 4 °C. The lysates were then centrifuged at 16,000× *g* for 20 min, and the supernatants were collected for further analysis. Protein samples (30 μg) were separated on 10% Mini-PROTEAN^®^ TGX™ Precast Gels (Bio-Rad Laboratories, Hercules, CA, USA) and transferred onto polyvinylidene difluoride membranes using the Trans-Blot Turbo Transfer System (Bio-Rad Laboratories). Membranes were blocked with 3% skim milk in Tris-buffered saline with 0.1% Tween 20 (TBST) for 2 h at RT, then incubated overnight at 4 °C with the following primary rabbit antibodies diluted in blocking solution: monoclonal anti-CDKN2A/p16^INK4a^ IgG (1:100, Cell Signaling Technology, Danvers, MA, USA, Cat. No. 18769T), monoclonal anti-phospho-γH2AX (Ser139) IgG (1:1000, Thermo Fischer Scientific, Waltham, MA, USA, Cat. No. MA5-44300), monoclonal anti-acetyl-p53 (Lys370) (Ac-p53) IgG (1:100, Abcam, Cambridge, UK, Cat. No. ab183544), and monoclonal anti-GAPDH IgG (1:2000, Cell Signaling Technology, Danvers, MA, USA, Cat. No. 2118). After three TBST washes, membranes were incubated for 1 h at 37 °C with secondary goat anti-rabbit IgG H&L conjugated with horseradish peroxidase (HRP) (1:2000, Abcam, Cambridge, UK, Cat. No. ab6721). Following three additional washes, protein bands were visualized using an enhanced chemiluminescence detection system (ImmunoStar LD, Wako Pure Chemical Industries, Osaka, Japan) and imaged with an ImageQuant LAS4000mini chemiluminescence imager (GE Healthcare, Chicago, IL, USA). Band intensity was quantified using the Image J 1.51f software.

### 2.9. Statistical Analyses

Data are expressed as pooled means + standard error of the mean (+S.E.M.) from at least three independent biological experiments. The number of independent biological replicates (*n*) is indicated in the corresponding figure legends and reflects separately performed experiments rather than technical replicates. Statistical analyses were performed using GraphPad Prism 7.05 software (GraphPad Software, La Jolla, CA, USA). Comparisons among multiple experimental groups were analyzed using one-way analysis of variance (ANOVA) followed by Dunnett’s multiple-comparisons post hoc test. A *p*-value < 0.05 was considered statistically significant.

## 3. Results

### 3.1. Melatonin Attenuates UVB-Induced Loss of Keratinocyte Viability and Proliferative Capacity

**Figure 1:**
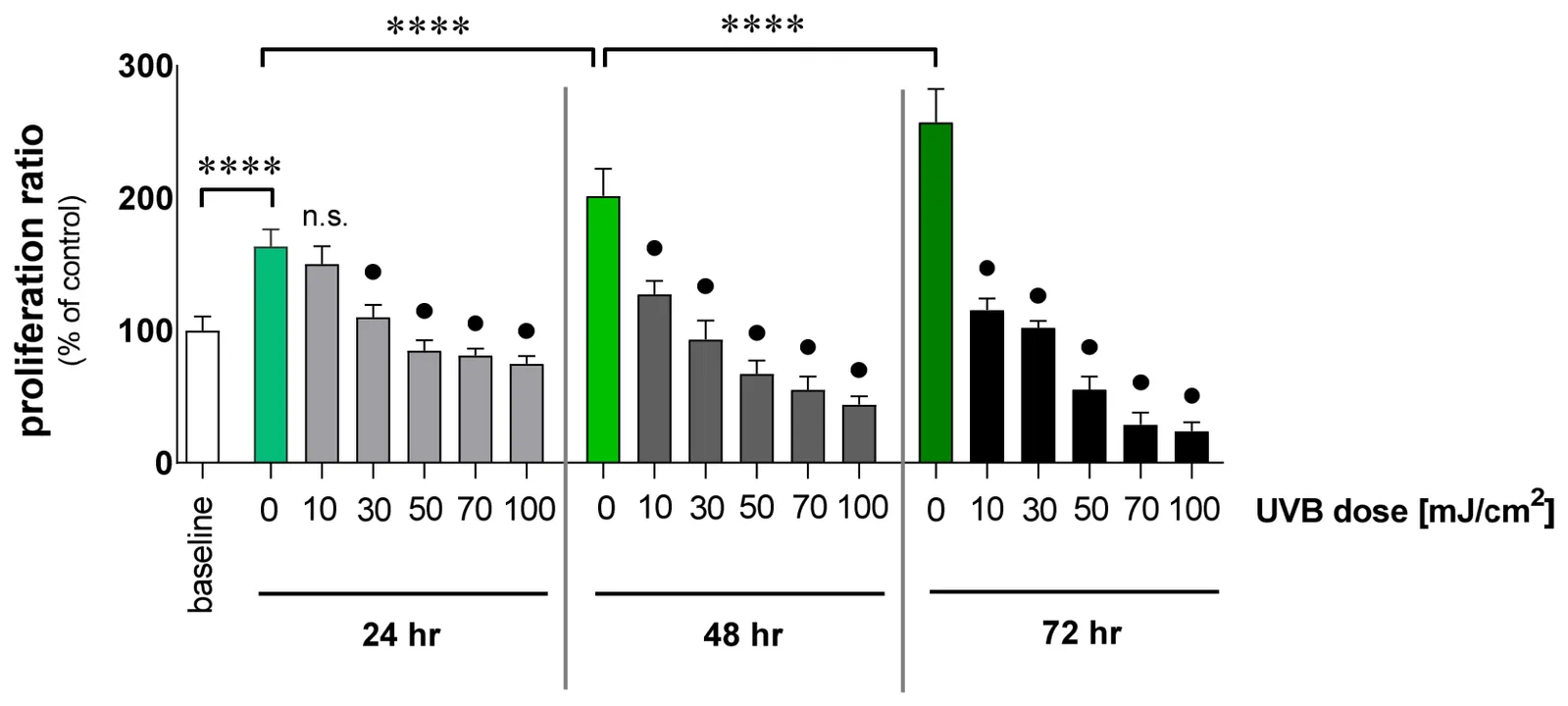
UVB irradiation induces a dose-dependent reduction in keratinocyte viability. Cells were exposed to increasing doses of UVB (0–100 mJ/cm^2^), and cell viability was assessed using the MTT assay as described in Materials and Methods. The “baseline” bar represents untreated, sham-irradiated keratinocytes assessed at the initial reference time point, whereas the first bar in each time-course (0 mJ/cm^2^) represents sham-irradiated control keratinocytes cultured for the corresponding experimental time point (24, 48, or 72 h; green bars). Data are presented as the mean + S.E.M. of four independent biological experiments (*n* = 4) and are expressed as a percentage of the corresponding sham-irradiated groups is as **** *p* < 0.0001, whereas differences between UVB-exposed cells and the corresponding sham-irradiated controls at each experimental time point (24, 48, or 72 h) are indicated as • *p* < 0.001. n.s.—not significant. The progressive increase in the sham-irradiated groups reflects normal cell growth during the culture period.

To evaluate the cytoprotective effects of melatonin against UVB-induced damage, human epidermal keratinocytes were exposed to increasing doses of UVB radiation and cell viability was assessed using complementary metabolic (MTT) and lysosomal integrity (Neutral Red, NR) assays. Namely, UVB irradiation resulted in a clear dose-dependent decline in keratinocyte viability, with a pronounced (*p* < 0.0001) reduction observed at 30 mJ/cm^2^ (24 h) and 10 mJ/cm^2^ (48 h and 72 h post-UVB) and higher doses, indicating substantial cellular stress and compromised metabolic activity (Figure 1).

**Figure 2:**
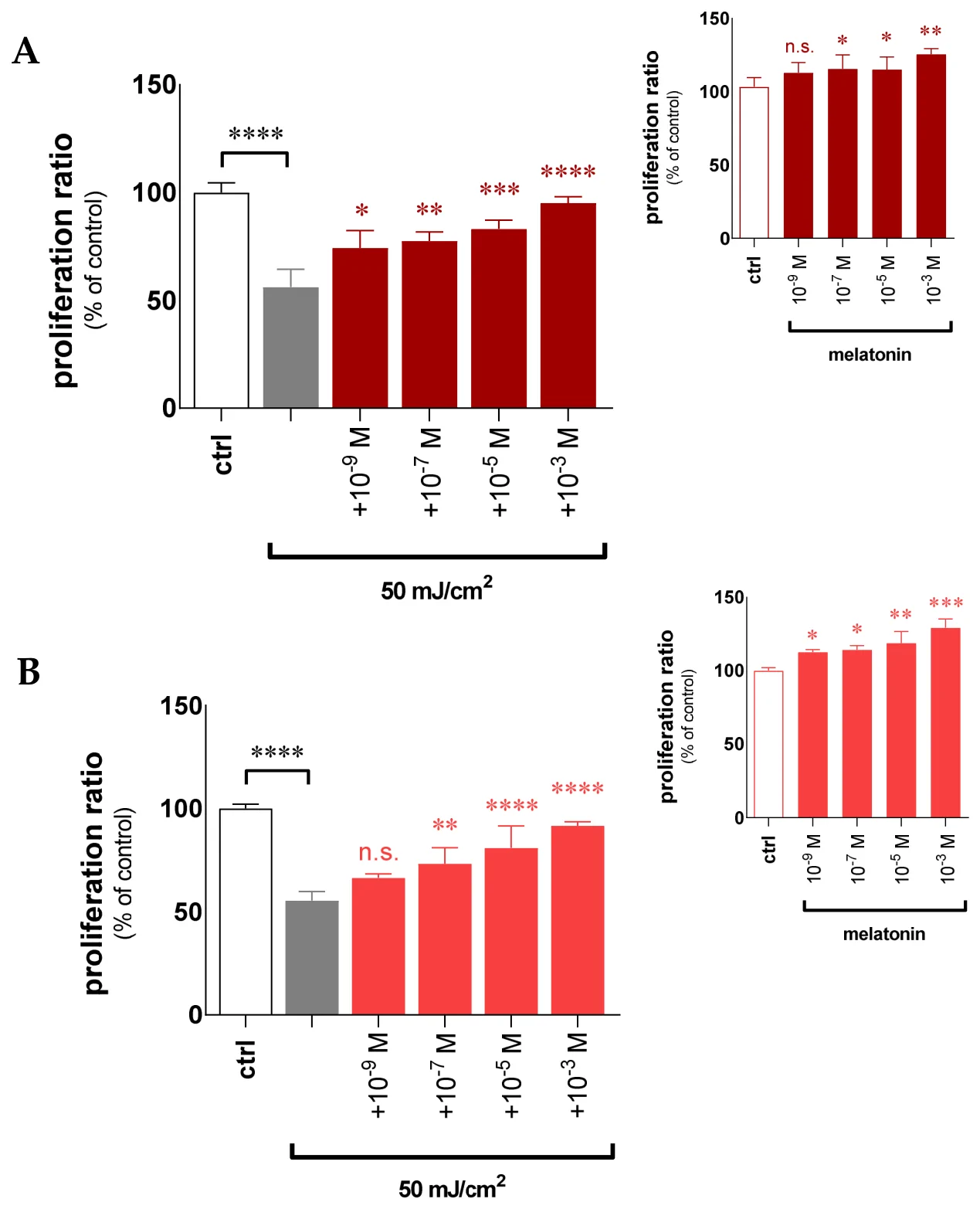
Melatonin preserves keratinocyte viability following UVB exposure. Cells were pre-incubated with melatonin (10^−9^, 10^−7^, 10^−5^, or 10^−3^ M) for 1 h and subsequently exposed to UVB irradiation (50 mJ/cm^2^), and proliferation ratio was assessed 48 h post-irradiation. Cell viability was evaluated using the MTT assay (**A**) and the Neutral Red (NR) uptake assay (**B**) as described in Materials and Methods. Data are presented as the mean + S.E.M., (*n* = 4) and expressed as a percentage of sham-irradiated control cells (0 mJ/cm^2^). Statistically significant differences between UVB-exposed cells and control sample are indicated as **** *p* < 0.0001 (black asterisks), statistical differences (colour asterisks) compare samples between UVB + melatonin and UVB alone as * *p* < 0.05, ** *p* < 0.01, *** *p* < 0.001, **** *p* < 0.0001. n.s.—not significant. *Inlets* show the proliferating effect of melatonin by enhanced metabolic activity and lysosomal integrity.

In sham-irradiated cultures, the MTT signal increased by approximately 63.51%, 101.63% and 157.35% after 24, 48, and 72 h, respectively, relative to the initial control value (baseline). This increase reflects the normal time-dependent growth of subconfluent keratinocyte cultures and the corresponding increase in metabolic activity during the culture period rather than an effect of the sham irradiation procedure. Furthermore, pre-incubation in dose-dependent manner with melatonin significantly mitigated UVB-induced cytotoxicity across a range of concentrations. Notably, melatonin showed slight protective effect at the dose of 10^−9^ M (18.11%, *p* < 0.05) and 10^−7^ M (21.40%, *p* < 0.01) and the most effective concentration was found for 10^−3^ M (38.99%, *p* < 0.0001) compared to UVB-exposed cells where the cell viability was maintained, partially restoring both mitochondrial dehydrogenase activity (MTT assay) (Figure 2A). Similar pattern of regulation was noticed in case of lysosomal function (NR assay) (Figure 2B).

**Figure 3:**
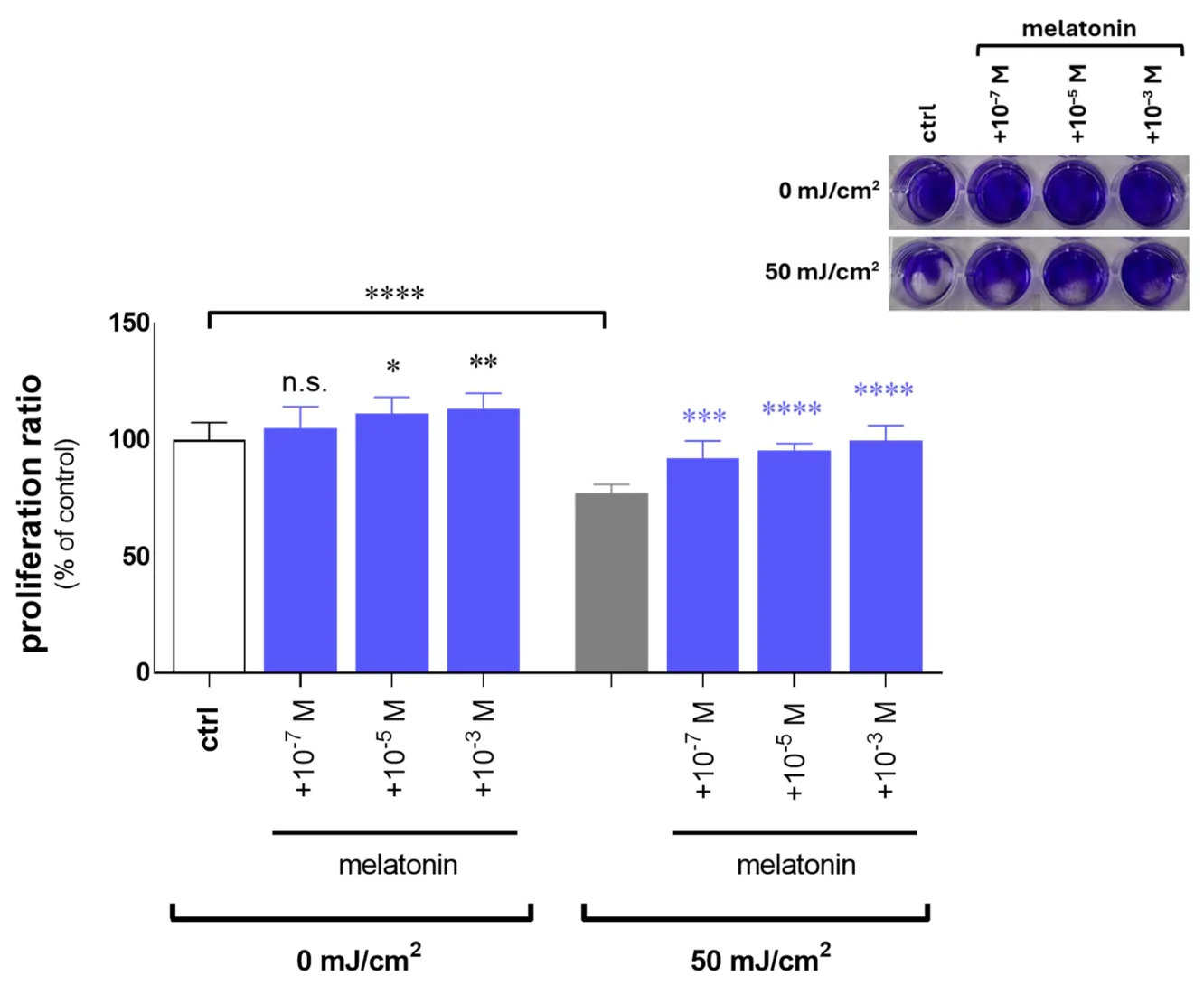
Melatonin preserves keratinocyte adherence and cell density following UVB irradiation. Adherent keratinocyte cell density was evaluated using crystal violet staining following pre-treatment with melatonin (10^−7^, 10^−5^, or 10^−3^ M) for 1 h and subsequent UVB exposure (50 mJ/cm^2^). Representative images and quantitative analysis are shown. Stained cell density was quantified using ImageJ 1.54j software and normalized to sham-irradiated control cells. Data are presented as the mean + S.E.M., (*n* = 5) and statistically significant differences versus control cells are indicated as * *p* < 0.05, ** *p* < 0.01, **** *p* < 0.0001 (black asterisks) and differences between UVB + melatonin and UVB alone as *** *p* < 0.001, **** *p* < 0.0001 (colored asterisks). n.s.—not significant.

It should be noted that lower or higher concentrations of melatonin exhibited reduced efficacy, suggesting a concentration-dependent protective profile. Importantly, melatonin alone did not reduce keratinocyte viability under non-irradiated conditions. At higher concentrations (10^−5^–10^−3^ M), a modest but statistically significant increase in MTT and NR signals was observed, consistent with enhanced metabolic activity and/or viable cell number rather than cytotoxicity (Figure 2, inlets). In accordance with these findings, crystal violet staining revealed that UVB exposure markedly reduced adherent cell density (*p* < 0.0001), reflecting decreased cell survival and/or retention following UVB-induced injury. Melatonin pre-treatment partially restored adherent cell density, supporting its cytoprotective effect in maintaining keratinocyte integrity following UVB exposure (Figure 3). Briefly, these data demonstrate that melatonin effectively counteracts UVB-induced impairment of keratinocyte viability and proliferative integrity. Based on these dose–response experiments, a UVB dose of 50 mJ/cm^2^ was selected for all subsequent mechanistic analyses because it produced an approximately 50% reduction in cell viability while preserving a sufficient number of viable cells for downstream biochemical and molecular assessments.

### 3.2. Melatonin Preserves Mitochondrial Redox Balance, Membrane Polarization, and Bioenergetic Capacity in UVB-Exposed Keratinocytes

Because mitochondrial dysfunction represents a key downstream consequence of UVB-induced oxidative stress and is closely linked to the development of mitochondrial dysfunction–associated senescence (MiDAS), we next assessed whether melatonin modulates mitochondrial redox status, membrane integrity, and energy production in irradiated keratinocytes.

**Figure 4:**
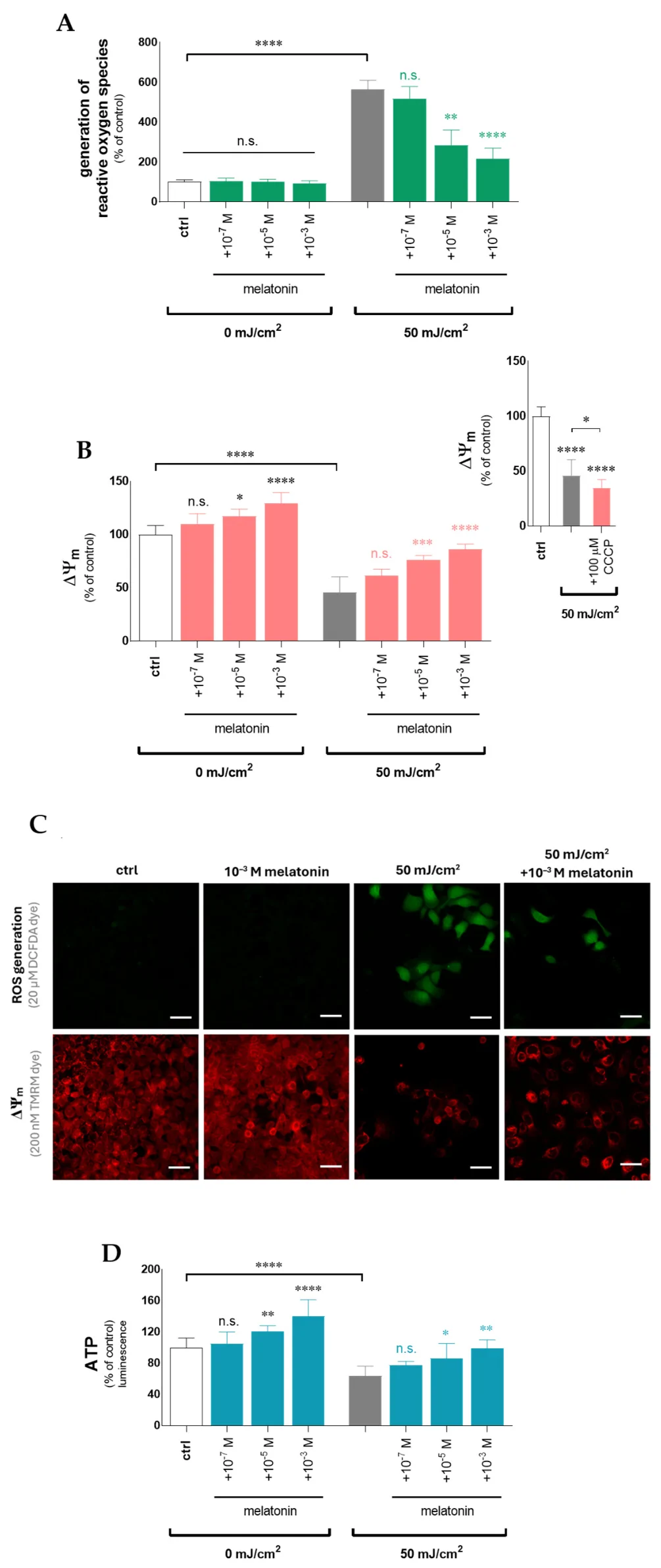
Melatonin suppresses UVB-induced oxidative stress and preserves mitochondrial membrane potential. Intracellular reactive oxygen species (ROS) generation (**A**) and mitochondrial transmembrane potential (ΔΨm) (**B**) were assessed in keratinocytes pre-treated for 1 h with melatonin (10^−3^ M) and exposed to UVB irradiation (50 mJ/cm^2^). ROS levels were measured using the DCFDA fluorescent probe (20 μM final concentration), while ΔΨm was evaluated using TMRM staining (200 nM final concentration), as described in Materials and Methods. Additionally, carbonyl cyanide m-chlorophenylhydrazone (100 μM CCCP) was used for 60 min as an independent positive control for ΔΨm depolarization (inlet) and was not subjected to UVB irradiation. Representative fluorescence microscopy images and quantitative analyses are shown (**C**), bars = 40 μm. Simultaneously, intracellular ATP levels were quantified using a luminescence-based ATP assay (**D**). Data are expressed as the mean + S.E.M., (*n* = 4) and normalized to sham-irradiated control cells. Statistically significant differences versus control cells are indicated as * *p* < 0.05, ** *p* < 0.01, **** *p* < 0.0001 (black asterisks) and differences between UVB + melatonin and UVB alone as * *p* < 0.05, ** *p* < 0.01, *** *p* < 0.001, **** *p* < 0.0001 (colored asterisks). n.s.—not significant.

UVB irradiation (50 mJ/cm^2^) dramatically increased intracellular reactive oxygen species (ROS) compared with sham-irradiated controls, reflecting elevated oxidative burden and disruption of mitochondrial redox homeostasis (Figure 4A). Contrary, pre-treatment with melatonin (10^−7^–10^−3^ M) markedly attenuated UVB-induced ROS accumulation, reducing fluorescence intensity toward baseline levels. Additionally, fluorescence microscopy corroborated these findings, revealing diminished ROS-associated signals in melatonin-treated keratinocytes (Figure 4C). Melatonin alone did not significantly affect basal ROS production. Given the sensitivity of mitochondrial membrane potential to oxidative stress, we noticed a pronounced depolarization of mitochondrial transmembrane potential (ΔΨm) after UVB exposure by 54.06% compared to control cells (Figure 4B).

In contrast, melatonin pre-treatment partially preserved ΔΨm following UVB irradiation. The effect was not statistically significant at 10^−7^ M (15.47%), but reached statistical significance at 10^−5^ M (17.03%, *p* < 0.001) and 10^−3^ M (33.88%, *p* < 0.0001). This was additionally evidenced by sustained TMRM fluorescence (Figure 4C) and compared to sham-irradiated controls. Inlet of the treatment with the mitochondrial uncoupler (100 μM CCCP) confirmed the assay specificity and validated the depolarization patterns.

Moreover, to support whether preservation of mitochondrial redox balance and ΔΨm translated into functional metabolic outcomes, intracellular ATP levels (Figure 4D) were evaluated using a luminescence-based assay where UVB irradiation resulted in a significant reduction in ATP synthesis (36.05% compared to control cells, *p* < 0.0001), which is consistent with impaired cellular ATP production, likely reflecting mitochondrial dysfunction–associated senescence (MiDAS). Treatment with melatonin before the irradiation significantly counteracted the UVB-induced decline in ATP levels, maintaining intracellular ATP content at the level of 77.51% (10^−7^ M), 85.91% (10^−5^ M) and 93.40% (10^−3^ M). Under non-irradiated conditions, melatonin alone produced a modest but statistically significant increase in intracellular ATP levels at 10^−5^ M and 10^−3^ M, whereas no reduction in ATP production was observed at any concentration tested. This is in line with the same pattern of regulation observed for ΔΨm (Figure 4B) as well as changes noticed at the level of proliferation assessments (Figure 2A,B; inlets and Figure 3). Collectively, these findings demonstrate that melatonin preserves mitochondrial homeostasis in UVB-exposed keratinocytes, as evidenced by reduced ROS accumulation, maintenance of ΔΨm, and preservation of ATP synthesis.

### 3.3. Melatonin Reduces UVB-Induced Apoptotic Morphology and Caspase Activation

**Figure 5:**
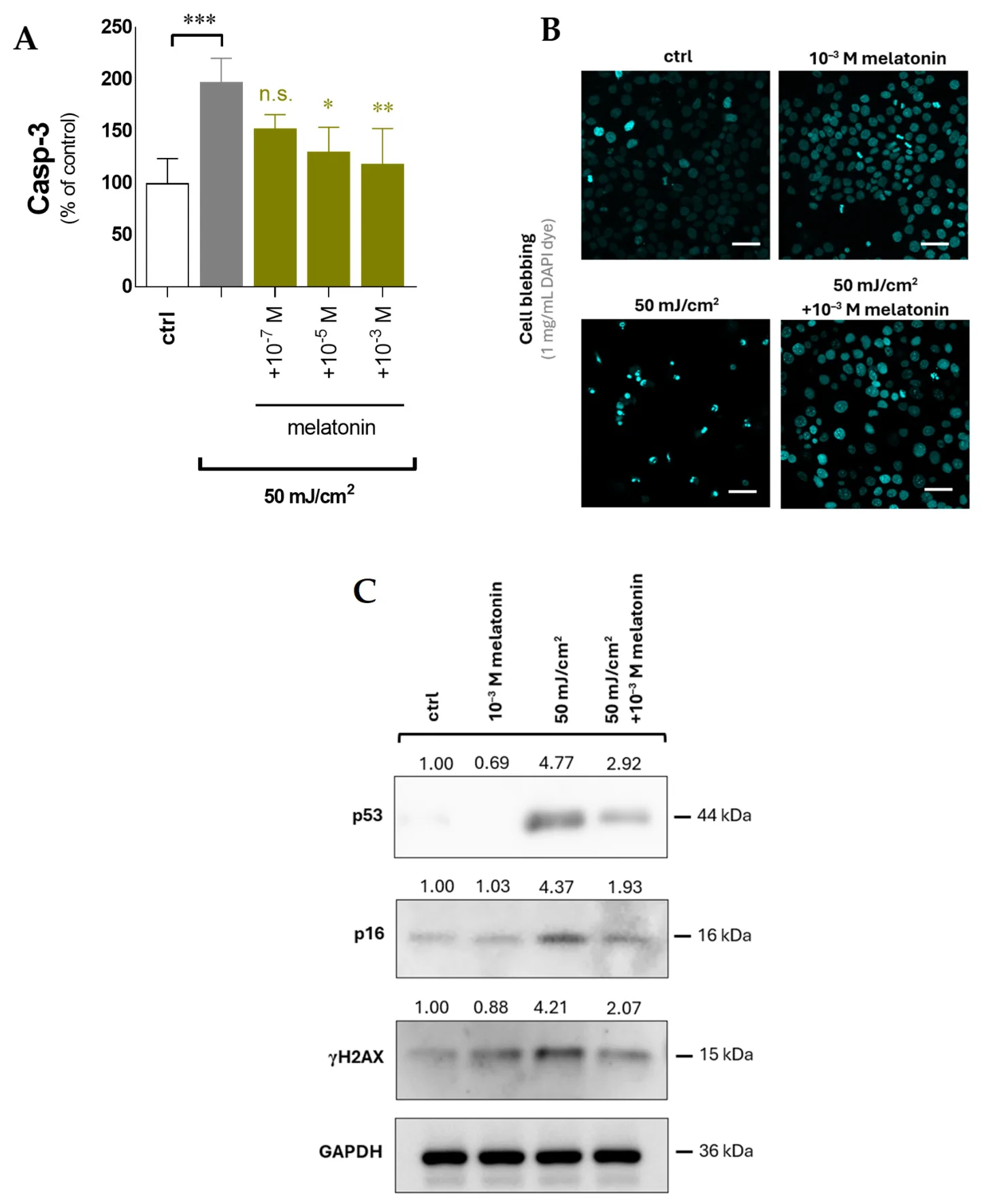
Melatonin attenuates UVB-induced apoptotic morphology and activation of DNA damage- and senescence-associated markers. (**A**) Caspase-3 activity was measured in keratinocytes following pre-treatment for 1 h with melatonin (10^−3^ M) and UVB exposure (50 mJ/cm^2^) using a luminescence-based assay as described in Materials and Methods. Data are expressed as the mean +S.E.M., (*n* = 6) and normalized to sham-irradiated control cells. Statistically significant differences between UVB-exposed cells are indicated as *** *p* < 0.001 (black asterisks) and differences between UVB + melatonin and UVB alone as * *p* < 0.05, ** *p* < 0.01 (colored asterisks). Representative fluorescence microscopy images showing nuclear morphology assessed by DAPI staining (**B**), bars = 40 μm. (**C**) Representative immunoblots are shown. Western blot analysis of Ac-p53, p16^INK4a^, and phosphorylated γH2AX protein expression following melatonin pre-treatment and UVB exposure where GAPDH was used as a loading control. Densitometric quantification relative to control conditions is shown above the respective blots. n.s.—not significant.

To assess whether melatonin-mediated preservation of mitochondrial bioenergetic function influences downstream cell death pathways, apoptotic features were evaluated morphologically and biochemically. Namely, UVB exposure significantly increased caspase-3 activity, indicating almost 2-fold activation of the executioner caspase cascade (Figure 5A) as direct down-stream consequences of the changes within the mitochondrial maintenance. On the other side, melatonin pre-treatment distinctly attenuated UVB-induced caspase activation by 45.12% (10^−7^ M), 67.43% (10^−5^ M) and 79.36% (10^−3^ M) compared to irradiated keratinocytes. Representative DAPI images qualitatively showed increased chromatin condensation and nuclear fragmentation following UVB irradiation (Figure 5B). These nuclear alterations were markedly reduced in keratinocytes pre-treated with melatonin. These results indicate that melatonin suppresses UVB-triggered apoptotic responses in keratinocytes, consistent with preservation of mitochondrial integrity and bioenergetic function.

### 3.4. Melatonin Attenuates UVB-Induced Activation of DNA Damage and Senescence-Associated Signaling Pathways

Because persistent mitochondrial dysfunction can promote sustained DNA damage responses and senescence-associated signaling, we examined whether melatonin modulates UVB-induced expression of key molecular markers linked to these processes. Western blot analysis revealed that UVB irradiation markedly increased phosphorylation of histone H2AX (γH2AX), indicating robust activation of DNA damage signaling pathways (Figure 5C). In addition, UVB exposure elevated protein levels of Ac-p53 and p16^INK4a^, consistent with activation of stress-responsive and cell cycle-regulatory pathways. On the other side, treatment with 10^−3^ M melatonin attenuated UVB-induced accumulation of γH2AX, indicating a reduction in DNA damage signaling. Besides, melatonin also modulated the UVB-induced expression of p53 and p16^INK4a^, partially suppressing their induction compared with UVB-irradiated cells. Importantly, melatonin treatment alone did not induce detectable changes in the expression of these markers under non-irradiated conditions. Herein, these data demonstrated that melatonin limits UVB-induced activation of DNA damage signaling and modulates the expression of proteins associated with senescence-related pathways in human keratinocytes.

### 3.5. Integrated Mitochondrial-Centered Protective Effects of Melatonin in UVB-Exposed Keratinocytes

Collectively, the in vitro data demonstrate that melatonin exerts coordinated protective effects against UVB-induced cellular stress in human epidermal keratinocytes. Namely, melatonin preserves cell viability and proliferative capacity, suppresses intracellular oxidative stress, stabilizes mitochondrial membrane potential, and maintains ATP synthesis following UVB exposure. In parallel, melatonin attenuates UVB-induced apoptotic signaling and limits activation of DNA damage- and senescence-associated molecular markers.

**Figure 6:**
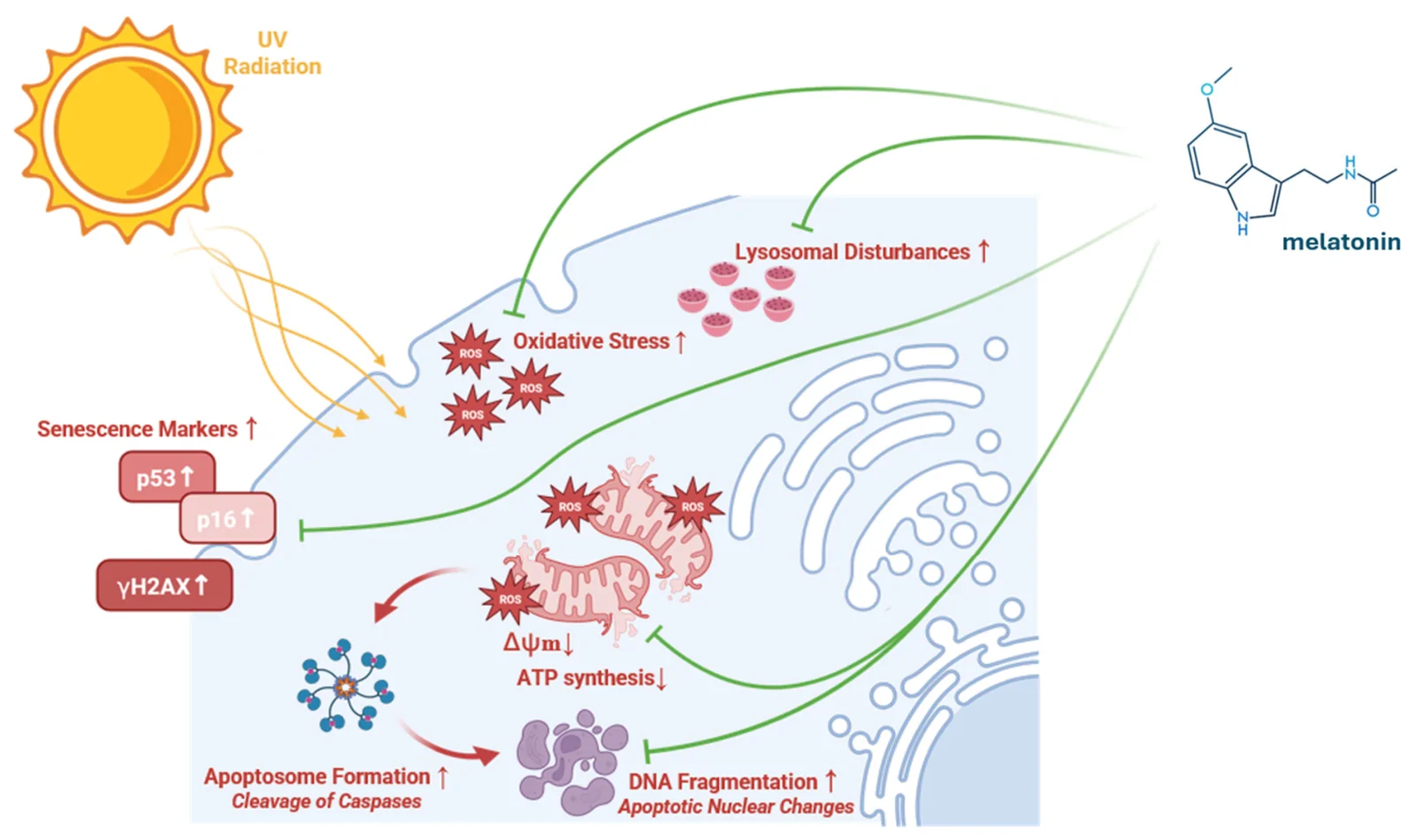
Schematic summary of mitochondrial-centered protective actions of melatonin in UVB-exposed keratinocytes. Schematic illustration summarizing the effects of melatonin on UVB-induced cellular stress in human epidermal keratinocytes. Namely, melatonin is reported to suppress oxidative stress, preserves mitochondrial membrane potential (ΔΨm), maintains ATP synthesis, and attenuates activation of apoptotic and senescence-associated signaling pathways, supporting mitochondrial function and cellular homeostasis following UVB exposure.

Taken together, these findings are consistent with mitochondrial homeostasis representing a major downstream target of melatonin action under UVB-induced stress and suggest that preservation of mitochondrial redox balance and bioenergetic capacity is associated with reduced downstream activation of apoptotic and senescence-related stress responses in UVB-irradiated keratinocytes (Figure 6). Importantly, these mechanistic insights provide a foundation for our future in vivo studies aimed at evaluating whether the role of melatonin in age-associated skin changes under physiological aging conditions.

## 4. Discussion

Ultraviolet radiation is a major extrinsic component of the cutaneous exposome and a key driver of epidermal damage, photoaging, and carcinogenesis through mechanisms involving oxidative stress, mitochondrial dysfunction, and persistent activation of DNA damage responses [43,44,45,46,47]. In the present study, we demonstrate that melatonin effectively protects human epidermal keratinocytes against UVB-induced cellular stress by preserving mitochondrial redox balance, maintaining bioenergetic capacity, and attenuating downstream apoptotic and senescence-associated signaling. These findings place mitochondrial homeostasis at the center of melatonin’s cytoprotective actions in the epidermis and integrate well into emerging concepts that skin aging is under tight endocrine and neuroendocrine regulation [31,37,48].

It is well-documented that human skin possesses an autonomous melatoninergic system capable of synthesizing melatonin and its biologically active metabolites locally, independent of pineal secretion [31,49,50,51,52,53,54]. This system includes enzymatic machinery for melatonin biosynthesis and metabolism, receptor-dependent and receptor-independent signaling pathways, and generates multiple metabolites with potent antioxidant and cytoprotective properties [38,55,56,57,58,59]. Although both receptor-dependent (MT1/MT2-mediated) and receptor-independent mechanisms have been proposed to contribute to melatonin’s biological actions, the present study was not designed to distinguish between these signaling pathways. Consequently, our findings should be interpreted as demonstrating the overall cytoprotective effects of melatonin on mitochondrial homeostasis rather than defining the relative contribution of specific receptor-mediated or receptor-independent mechanisms. Future studies employing selective receptor antagonists, genetic approaches, or pathway-specific inhibitors will be required to clarify these mechanisms.

Importantly, melatonin and its metabolites accumulate in cutaneous compartments at concentrations exceeding circulating plasma levels, underscoring their role as locally acting regulators of skin homeostasis rather than classical endocrine hormones [37,60,61].

The concentration range used in the present study should also be considered in the context of the cutaneous melatonergic system. The lower melatonin concentrations tested (10^−9^–10^−9^ M) are closer to tissue-relevant levels reported for cutaneous melatonin synthesis and metabolism [31,32,33,34]. In contrast, the highest concentration (10^−3^ M) represents a supraphysiological pharmacological condition that was included to define the upper boundary of cytoprotective efficacy under severe UVB-induced stress. Accordingly, the more pronounced protective effects observed at 10^−3^ M should be interpreted as a pharmacological ceiling effect rather than a direct reflection of endogenous melatonin concentrations in human skin. Nevertheless, the protective responses observed at lower concentrations further support the physiological relevance of melatonin-mediated cytoprotection in the epidermis. On the other side, UVB irradiation is a potent inducer of oxidative stress in keratinocytes, is associated with increased reactive oxygen species (ROS) production, mitochondrial membrane depolarization, and impaired oxidative phosphorylation. In agreement with previous studies [4,7,38,48,58,59,62] demonstrating that melatonin suppresses UV-induced ROS generation, oxidative DNA damage, and depletion of antioxidant enzyme activity in keratinocytes and human skin, our data confirm that melatonin markedly reduces UVB-induced mitochondrial alterations. By directly linking melatonin treatment to preservation of mitochondrial bioenergetic function, our findings are consistent with mitochondrial homeostasis representing a major downstream target of melatonin action in UVB-exposed epidermal cells. This mitochondrial focus is particularly relevant in light of recent high-impact syntheses emphasizing mitochondria as integrative hubs where endocrine signals, redox balance, and metabolic state converge to regulate skin aging [31,33,37,43,63]. Thus, melatonin’s amphiphilic nature allows efficient penetration into mitochondria, where it acts not only as a direct free-radical scavenger but also as a stabilizer of mitochondrial membranes and electron transport chain function [31,33,47,48]. Moreover, mitochondria have been proposed as sites of melatonin synthesis and metabolism, generating downstream indolic and kynuric metabolites that retain antioxidant activity and further reinforce mitochondrial protection [33,34,52,53,54,55,56,57,58,59,64]. Moreover, persistent mitochondrial dysfunction is increasingly recognized as a critical driver of DNA damage responses, apoptosis, and cellular senescence in the skin. UV-induced phosphorylation of histone H2AX (γH2AX) reflects activation of DNA damage signaling pathways that may persist long after irradiation and contribute to premature skin aging. Previous studies in UVA-exposed HaCaT cells demonstrated sustained γH2AX accumulation indicative of prolonged genomic stress rather than transient repair events [65,66,67]. Our results extend these observations to UVB exposure, showing robust induction of γH2AX alongside increased expression of p53 and p16^INK4a^, markers associated with stress-induced cell-cycle arrest and senescence initiation. In our report, representative immunoblot analysis indicated that melatonin reduces UVB-induced γH2AX accumulation and modulated p53 and p16^INK4a^ expression, indicating reduced activation of DNA damage and senescence-associated signaling. Although these markers alone do not define a fully senescent phenotype, their modulation is consistent with attenuation of early senescence-related signaling events commonly observed in UV-stressed keratinocytes and aged skin [68,69]. It should be noted that recent conceptual advances have highlighted mitochondrial dysfunction-associated senescence (MiDAS) as a senescence program driven primarily by impaired mitochondrial function, altered bioenergetics, and mitochondrial ROS signaling rather than irreversible nuclear damage alone [45,70,71,72]. Indeed, hallmarks of MiDAS include mitochondrial depolarization, reduced ATP production, and metabolic reprogramming. By preserving mitochondrial membrane potential and ATP synthesis, our findings indicate that melatonin attenuates several features associated with MiDAS. These observations are consistent with an association between preservation of mitochondrial homeostasis and reduced senescence-associated signaling in UVB-stressed keratinocytes. Our findings further support the concept that mitochondrial homeostasis is closely associated with cellular responses to UV-induced stress, including apoptotic signaling and early senescence-related events. In line with earlier reports demonstrating anti-apoptotic effects of melatonin in UV-exposed keratinocytes and human skin explants [31,35,37,43,48], our data show that melatonin attenuates UVB-induced caspase-3 activation and apoptotic morphology. Finally, these findings suggest that melatonin reduces overall stress intensity rather than enforcing a specific cellular outcome, which is associated with preserving keratinocyte viability and functional integrity. Several limitations of the present study should be acknowledged. Although the present findings consistently demonstrate the cytoprotective effects of melatonin in UVB-exposed keratinocytes, the relative contributions of receptor-dependent and receptor-independent signaling pathways were not investigated. In addition, the highest melatonin concentration (10^−3^ M) represents a pharmacological rather than physiological exposure, and no independent antioxidant comparator was included. Finally, the Western blot results are presented as representative immunoblots without quantitative densitometric analysis. Although the observed expression patterns were consistent with the remaining experimental findings, future studies incorporating quantitative analysis of multiple independent immunoblots and mechanistic approaches will further strengthen these observations.

## 5. Conclusions

Collectively, the present study demonstrates that melatonin exerts potent protective effects against UVB-induced cellular stress in human epidermal keratinocytes by preserving mitochondrial homeostasis. Melatonin maintains mitochondrial redox balance, preserves mitochondrial membrane potential and bioenergetic capacity, and attenuates the activation of apoptotic and DNA damage-associated signaling pathways following UVB exposure. By stabilizing mitochondrial function, melatonin limits key upstream events involved in oxidative stress amplification, mitochondrial dysfunction, and the early activation of senescence-associated pathways.

These findings further support mitochondrial homeostasis as a major cellular target of melatonin and provide new mechanistic insight into the role of the cutaneous melatonergic system in maintaining epidermal resilience to ultraviolet stress. An important limitation of the present study is that it was conducted exclusively using an in vitro UVB-induced keratinocyte model. Nevertheless, the results establish a strong foundation for future studies in more physiologically relevant systems. Future in vivo investigations will determine whether the mitochondrial protective mechanisms identified here translate into reduced oxidative damage, preservation of skin homeostasis, and attenuation of molecular markers of photoaging following UVB exposure.

## Data Availability

The original contributions presented in this study are included in the article. Further inquiries can be directed to the corresponding author.
